# Studying highly nonlinear oscillators using the non-perturbative methodology

**DOI:** 10.1038/s41598-023-47519-5

**Published:** 2023-11-20

**Authors:** Galal M. Moatimid, T. S. Amer, A. A. Galal

**Affiliations:** 1 Department of Mathematics, Faculty of Education, Ain Shams University, Cairo, Egypt; 2 Department of Mathematics, Faculty of Science, Tanta University, Tanta, 31527 Egypt; 3 Department of Engineering Physics and Mathematics, Faculty of Engineering, Tanta University, Tanta, 31734 Egypt

**Keywords:** Engineering, Mechanical engineering

## Abstract

Due to the growing concentration in the field of the nonlinear oscillators (NOSs), the present study aims to use the general He's frequency formula (HFF) to examine the analytical representations for particular kinds of strong NOSs. Three real-world examples are demonstrated by a variety of engineering and scientific disciplines. The new approach is evidently simple and requires less computation than the other perturbation techniques used in this field. The new methodology that is termed as the non-perturbative methodology (NPM) refers to this innovatory strategy, which merely transforms the nonlinear ordinary differential equation (ODE) into a linear one. The method yields a new frequency that is equivalent to the linear ODE as well as a new damping term that may be produced. A thorough explanation of the NPM is offered for the reader's convenience. A numerical comparison utilizing the Mathematical Software (MS) is used to verify the theoretical results. The precise numeric and theoretical solutions exhibited excellent consistency. As is commonly recognized, when the restoration forces are in effect, all traditional perturbation procedures employ Taylor expansion to expand these forces and then reduce the complexity of the specified problem. This susceptibility no longer exists in the presence of the non-perturbative solution (NPS). Additionally, with the NPM, which was not achievable with older conventional approaches, one can scrutinize examining the problem's stability. The NPS is therefore a more reliable source when examining approximations of solutions for severe NOSs. In fact, the above two reasons create the novelty of the present approach. The NPS is also readily transferable for additional nonlinear issues, making it a useful tool in the fields of applied science and engineering, especially in the topic of the dynamical systems.

## Introduction

Various fields use linear and nonlinear differential equations (DEs) to express numerous problems related to mathematics, physics, biology, chemistry, and engineering. In contrast to nonlinear DEs, which were frequently assumed to have approximate solutions by using several perturbation approaches, the solutions to a linear DE can be naturally determined utilizing a few of firmly established techniques. Furthermore, since the majority of vibration problems are nonlinear, the nonlinear oscillations have attracted the attention of more and more scientists. Therefore, because scientific and engineering phenomena frequently take the form of nonlinear types, the nonlinear differential equations (NLDEs) were extremely effective in describing these phenomena. Consequently, nonlinear oscillatory DEs were essential in applied mathematics, physics, and engineering^1^. In multiple research works of literature that deal with NLDEs that appear in diverse scientific and engineering disciplines, it was fundamental to highlight the importance of mathematical computations^2^. Many NLDEs can be numerically analyzed, but only a few of them can be solved directly. In order to determine the interaction between the amplitude and frequency of the NOSs, numerous approximate techniques have been used in the available literature. The most flexible tool in analyzing nonlinear engineering problems was the perturbation approach, which was frequently used to compute approximate analytic solutions of NLDEs^3–5^. Analytical approaches for nonlinear issues have garnered escalating interest among scientists and professionals in engineering due to the nonlinear sciences' rapid development during the past two decades. It was developed to study the behavior of these NLDEs using numerical and other approximation methods^6–14^. There have been a number of new techniques for finding analytical solutions to the NLDE recently. Therefore, many researchers developed a few novel methods. For the purpose of obtaining analytical responses that are relatively proximate to the precise solutions, several scientists have investigated a variety of novel and distinctive methodologies. The Lindstedt-Poincaré approach^15^, the iterative perturbation approach^16^, and the homotopy perturbation method (HPM)^17–21^ are a few of these techniques.

In order to transform a nonlinear equation into one that is a linear one, the HFF was developed. A review of several recent asymptotic approach advancements applied to strongly and weakly nonlinear equations was examined^22^. Moreover, the approximations of the solutions were valid across the whole time interval. In response to the limitations of the perturbation approaches, numerous adapted strategies were introduced, in combination of mathematical tools like the theory of variational, methodology of the homotopy, and iteration techniques. A quick estimation of a NOS's periodic characteristic was crucial for engineering. The discussion of some of the simplest approaches for NOSs included the HFF, the max–min strategy, and the HPM^23^. This frequency was mathematically clarified, incorporating a weighted average to enhance the accuracy of the predicted frequency. A severe of NOS using a straightforward and excused technique was studied^24^. The simplest calculation may be used to promptly determine its frequency quality. The results showed that the approach yielded a response of an approximately accurate nature. This relationship was vital in the influence of designing a packaging system and a system of micro-electromechanical. It was revealed that the frequency of a nonlinear vibration system was fundamentally linked to its amplitude in a nonlinear manner. For NOSs with any beginning circumstances, this work offered a simple frequency prediction approach^25^. When the results were compared to those from the HPM, they showed a good level of agreement. An exceedingly practical technique for gaining an immediate and precise understanding of the nonlinear vibration of the system characteristics was developed. The discussion of a rotating pendulum, periodic properties, and instability qualities followed the application of He's frequency. The formula HFF was used to derive the governing NLDE of the analytical solution because there was frequently a perfect solution to a linear equation. The linearized equation, also known as a quasi-exact solution, represented an almost precise solution to the nonlinear equation. Whatever the case, solving a linear equation was simpler than solving a nonlinear equation. To cover the damping NOS, a developed He's frequency was constructed^26^. After converting the NOS to a linear damping model utilizing the conservative force of restoration, the resulting frequency from the odd nonlinear damping force was determined.

The existing study uses the NPM, which was recently created by El-Dib^27^, as a vital method to find the analytical approximation solutions to the three NLDEs. To show the effectiveness and validating accuracy of the current method, a comparison is made with the numerical solutions (NSs). It is obvious that, for the aforementioned issues, the current technique provides more accurate findings than comparable approximations. This occurs because of the very small absolute error when matching the NSs of the nonlinear DE and the linear one. The NPS has significant potential and may be used to solve other strongly nonlinear situations. In accumulation, the NPS resolves a number of real-world instances. These real-world instances were previously resolved using other established analytical techniques described in the literature. However, the current methodology yields best outcomes more quickly. However, with the NPS, the processes for identifying the approximate solutions are completely shown, and the computations using MS are even easier to perform than computations using other analytical techniques. In calculation, other approaches are difficult to apply or take a long time to analyze the solution. Regarding the adopted exclusive technique or significant results, it's important to emphasise the following outcomes:

1. The employed method yields an alternative equivalent linear differential equation, which is comparable to the existing nonlinear one.
2. Throughout this method, the two equations are perfectly matched to each other.
3. In the situations of involving the restoring forces, all the conventional methods are commonly employing Taylor expansion to simplify the complexity of the problem. However, this limitation is eliminated in the current approach.
4. Despite of the other conventional techniques, the NPM enables us to study the stability of the problem.
5. The original strategy appears to pick a straightforward, practical, and interesting tool. It has the capability to be applied to analyze numerous NOS categories.

To layout the presentation of the current work, there are five sections in the paper as follows. We introduce a rapid description of the NPM in “Description of NPM”. In “Applications”, the NPM is used to examine the three real-world NLDEs. Finally, “Conclusions” summarizes the closing observations.

## Description of NPM

The goal in this section is to replace the actual nonlinear structure with an alternative scheme that has established solutions that may be roughly approximated to the original system^28^. In other words, it is possible to convert a non-linear second-order DE to a linear one in an operation that minimizes the average of the difference between the two systems. The averaging operator holds specific properties, it is demonstrated that the replacement can be achieved straightforwardly. The fundamental principle of HFF is currently used to get the NOS into a linearized form, which results in a linear oscillator producing a solution that encompasses the entire time span of the oscillation history^29^. A generalized equivalent linear system's existence and uniqueness were previously subject to in-depth analysis^7^. Now, the NPM can be described as follows:

Within a given NLDE, a homogeneous third degree of nonlinear forces can be abstracted as including three distinct components: quadratic nonlinear damping forces, odd nonlinear damping forces, and finally the restoring nonlinear odd force. This implies that any NLDE can be restructured using these components, leading to the following exemplification:

1 $$\ddot{u} + f(u\,\dot{u},\ddot{u}) + g(u\,\dot{u},\ddot{u}) + h(u\,\dot{u},\ddot{u}) = 0,$$

where $f(u\,\dot{u},\ddot{u})$, are the odd secular terms, correspond to the Van der Pol-Rayleigh mechanism, $g(u\,\dot{u},\ddot{u})$, are the even non-secular terms, point to the quadratic nonlinearity of the Helmholtz appliance, and $h(u\,\dot{u},\ddot{u})$, are the odd secular terms, indicate cubic Duffing setup, in which they are classified as follows:

2 $$\begin{aligned} f(u,\,\dot{u},\,\ddot{u}) & = a_{1} \dot{u} + b_{1} u^{2} \dot{u} + c_{1} u\dot{u}^{2} + d_{1} \dot{u}^{3} + e_{1} \ddot{u}\dot{u}^{2} , \hfill \\ g(u,\,\dot{u},\,\ddot{u}) & = a_{2} \dot{u}u + b_{2} \dot{u}^{2} + c_{2} u^{2} + d_{2} \dot{u}\ddot{u}, \hfill \\ h(u,\,\dot{u},\,\ddot{u}) & = \omega^{2} u + b_{3} u^{2} \dot{u} + c_{3} u^{3} + d_{3} \ddot{u}u^{2} , \hfill \\ \end{aligned}$$

where $a_{j} ,\,b_{j} ,\,c_{j} ,\,d_{j} ,\,e_{j} \,\,(j = 1,\,2,\,3)$ are constant coefficients, and $\omega$ represents the natural frequency of the structure.

He's frequency^7^ has a simple structure that can be expanded upon to generate analytical expressions of the equivalent frequency $$\omega_{eqv}^{2}$$$$\Omega$$ for the damping oscillator of Helmholtz-Rayleigh-Duffing. For an easy-to-understand yet precise frequency-amplitude expression, the nearly equivalent linear oscillation described by Eq. (1) may be formulated relative to the nonlinear force of restoration $$\omega_{eqv}^{2} u$$, the force of damping $$\sigma \dot{u}$$, and the $$\lambda$$ inhomogeneity constant, as follows:

3 $$\ddot{u} + \sigma \dot{u} + \omega_{eqv}^{2} u = 0.$$

Equation (3) stands as a linear equation and can be resolved through conventional methodologies. The goal is to evaluate the coefficients featured in Eq. (3). Initially, assuming the loss of the damping constant $\sigma$ and the non-homogeneous part $\lambda$, the total frequency is simplified to the $\Omega^{2}$. The below harmonic equation is then obtained according to Eq. (3)

4 $$\ddot{u} + \Omega^{2} u = 0,$$

Equation (4) symbolizes the linear form of the simple harmonic oscillator. Lately, this issue has been examined by He^30^, utilizing the characteristics of special functions. The subsequent tentative solution is proposed as:

5 $$u = A\cos \Omega t.$$

Keeping in mind the starting conditions: $u(0) = A,\,\,\,{\text{and}}\,\,\,\,\dot{u}(0) = 0$.

Following El-Dib^27^, the three parameters that have arisen in Eq. (3) may be formulated as follows:

- Frequency formula
  The optimal approach for deriving the frequency formula involves employing the weighted residuals methodology. Employing He's formula proves beneficial in calculating frequencies for the higher generalized $h(u,\,\dot{u},\,\ddot{u})$. Approximating the frequency can be achieved by utilizing the weighted residuals method as introduced by El-Dib^27^ as
  6 $$\omega_{eqv}^{2} = \int\limits_{0}^{2\pi /\Omega } {uh(u,\,\dot{u},\,\ddot{u})} dt \bigg /\int\limits_{0}^{2\pi /\Omega } {u^{2} dt} .$$
- Damping formula
  The most effective method to derive the dampening formula is by using weighted residual techniques. You may calculate the frequency for specialized networks $f(u,\,\dot{u},\dddot u)$ using He's frequency. A preliminary estimate of the frequency can be considered as El-Dib^27^:
  7 $$\sigma = \int\limits_{0}^{2\pi /\Omega } {\dot{u}f(u,\,\dot{u},\,\ddot{u})} dt \bigg /\int\limits_{0}^{2\pi /\Omega } {\dot{u}^{2} dt} .$$
- Non-secular part
  It should be observed that the non-secular part has the quadratic formula. Therefore, the inhomogeneity will be computed by replacing: $u \to \frac{A}{2},\,\dot{u} \to \frac{A\Omega }{2},\,\,{\text{and}}\,\,\ddot{u} \to \frac{{A\Omega^{2} }}{2}$. It should be noted that this is true only up to the quadratic power.
  To this end, the nonlinear Eq. (1) is transformed into the linear one as given in Eq. (3). One can utilize the normal form of Eq. (3) to estimate the stability criteria in a simpler form, where the total frequency is determined from the formula: $\Omega^{2} = \omega_{eqv}^{2} - \sigma^{2} /4$.

## Applications

In this section, we propose the analytical analysis of a few real-world NLDEs so that you can evaluate the accuracy of NPM. The outcomes of the numerical methods are based on the analytically derived equivalent linear DEs. One can conclude from the outcomes that the NPM is more accurate than the other methods of the classical perturbations. Due to their critical role in understanding the complexities of several natural and physical occurrences as well as technological issues in numerous scientific domains, three instances are relevant for using this technique.

### First problem

**Figure 1:**
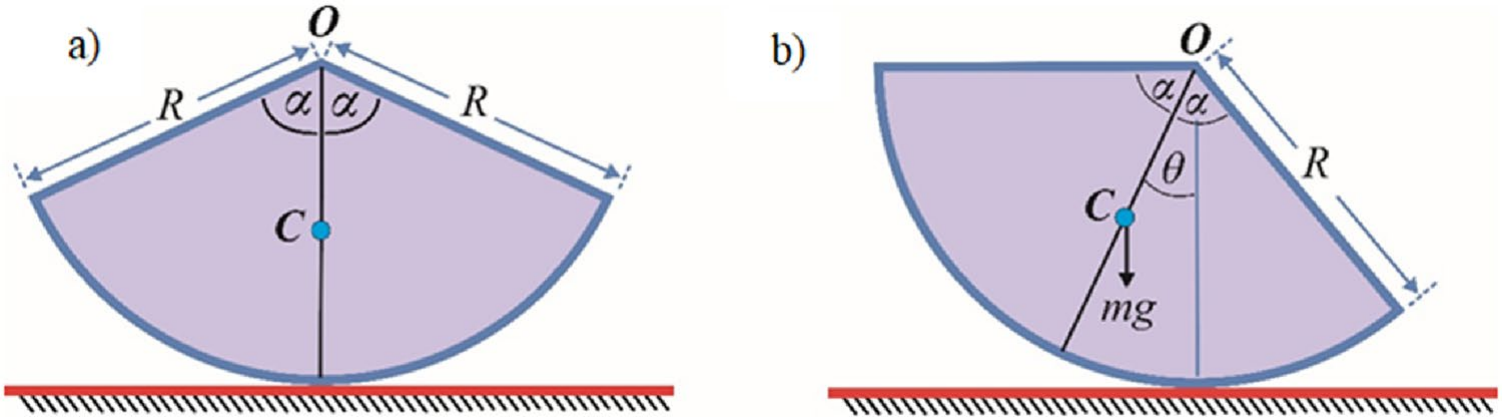
Illustrates the rotating problem of dynamic model.

A given symmetrical circular solid sector body with an angle of $\alpha$ and a radius of $R$ will be considered in the first application. These objects are frequently utilized in various physical and technical applications, such as automobile spaces and various swinging mechanisms. Therefore, the following problem may be formulated as follows:

A circular sector of a homogeneous solid cylinder is considered. Its centre of gravity is located at the point $C$. Its radius is assumed to be $R$, its central angle to be $2\alpha$ and its mass to be $m$. The sector is in a stationary state on a rough horizontal plane, as shown in Fig. 1a. It is subjected to an oscillation in the vertical plane, as illustrated in Fig. 1b. The motion here is under the influence of Earth's gravitational field. The last figure represents the actual position, where the rotation is signified by an angle of $\theta$ in the clockwise direction.

The derivation of the primary equation of motion can be summarized as follows:

The mass moment of the indicated sector about $C$ is evaluated as:

8 $$J_{C} = \frac{m}{2}R^{2} - m\,S^{2} ;\,\,\,\,\,\,\,S = \frac{2R}{3}\frac{\sin \alpha }{\alpha },$$

where $S$ is the distance from the centre of mass $C$ to the point $O$.

The kinetic energy of that sector may be written as:

9 $$T = \frac{m}{2}V_{C}^{2} - \frac{{J_{C} }}{2}\dot{\theta }^{2} ;\,\,\,\,\,\,\,\,\,\,\,V_{C}^{2} = \dot{\theta }^{2} (S^{2} + R^{2} - 2S\,R\cos \theta ),$$

where $V_{C}$ and $\dot{\theta }$ represent the velocity of the centre of gravity and the angular velocity, respectively.

The combination of Eqs. (8), (9) produce

10 $$T = \frac{m}{2}\dot{\theta }^{2} \left( {\frac{3}{2}R^{2} - 2R\,S\cos \theta } \right).$$

The stored potential energy of that system may be represented as follows:

11 $$P = mg\left( {R - S\cos \theta } \right).$$

The Equation of motion describing the oscillating sector is obtained directly from the Lagrange equation as given below:

12 $$\frac{d}{dt}\left( {\frac{dQ}{{d\dot{\theta }}}} \right) - \frac{dQ}{{d\theta }} = 0;\,\,\,\,Q = T - P.$$

Substituting Eqs. (10), (11) into (12), one gets

13 $$\left( {\frac{3}{2} - \frac{4}{3}\frac{\sin \alpha }{\alpha }\cos \theta } \right)R\ddot{\theta } + \frac{2R}{3}\frac{\sin \alpha }{\alpha }\dot{\theta }^{2} \sin \theta + \frac{2g}{3}\frac{\sin \alpha }{\alpha }\sin \theta = 0.$$

Inserting the transformation $\tau = \sqrt {g/R} \,t$ and the constant $k = \frac{4}{9}\frac{\sin \alpha }{\alpha }$ in Eq. (13), one obtains:

14 $$\theta^{\prime\prime} - 2k(\cos \theta )\theta^{\prime\prime} + k\theta^{{\prime}{2}} \sin \theta + k\sin \theta = 0\,;\,\,\,\,\,\,\,\left( {^{\prime} \equiv \frac{d}{d\tau }} \right).$$

It's important to mention that Eq. (14) is diverse from the equation that has been given by Amer et al.^31^ Actually, the variables are as they previously obtained. In contrast, the constant coefficients are completely different. Additionally, Ismail et al.^32^ are forced to expand the restoring forces by Taylor expansion, but the current NPM does not similar performance. Therefore, the pervious weakness will be ignored.

Based on the prior explanation of NPM, the basic equation of motion has the form:

15 $$\theta^{\prime\prime} + \psi (\theta ,\theta^{\prime},\theta^{\prime\prime}) = 0,$$

where $\psi (\theta ,\theta^{\prime},\theta^{\prime\prime}) = - 2k\theta^{\prime\prime}\cos \theta + k\theta^{{\prime}{2}} \sin \theta + k\sin \theta$ represents odd secular term, in which represents the cubic Duffing function.

Using analogous arguments as given throughout the previous section, the equivalent linear equation may be formulated as:

16 $$u^{\prime\prime} + \Omega^{2} u = 0,$$

where the corresponding frequency is given from an equation comparable with Eq. (6).

The equivalent frequency may be determined as follows using the Mathematica Software:

17 $${\Omega }^{2} = \frac{{2kJ_{1} (A)}}{{A - 4k J_{1} (A) + 2A k J_{2} (A)}},$$

where $J_{1} (A)$ and $J_{2} (A)$ are the Bessel functions.

For more convenience, it is interesting to examine the relationship involving the equivalent linear ODE that is obtained from the NPM and the NS of the previous Eq. (14) using the numerical calculations via Mathematica Software. As a consequence, the subsequent numbers for the applied settings are considered.

$$\alpha = \pi /6\;{\text{and}}\;A = 0.8.$$

**Figure 2:**
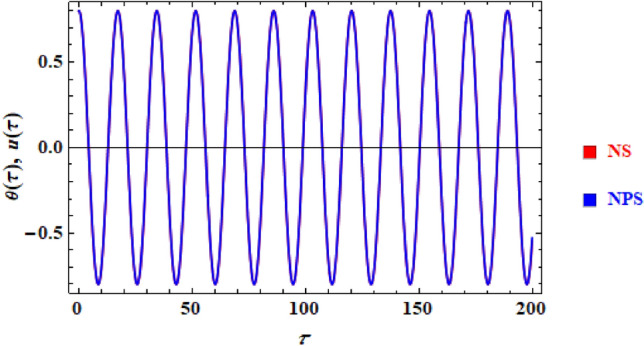
Displays a comparison between the NS and NPS.

**Figure 3:**
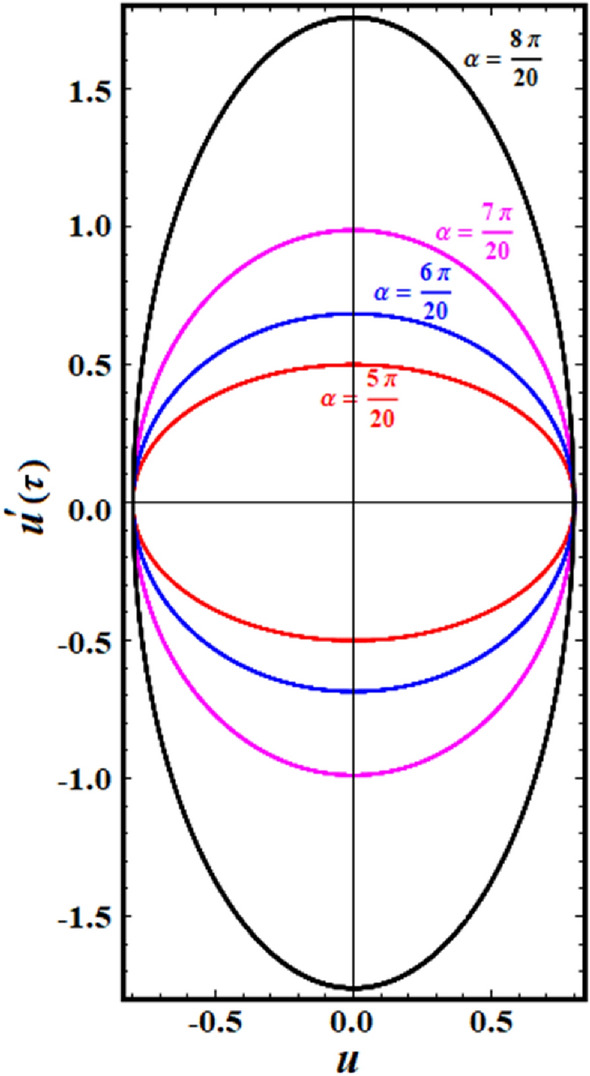
Presents the plots of phase plane for the NPS.

A comparison between the associated linear ODE equation and the NS of Eq. (15) produced by the numerical calculation is also useful. The non-perturbative equation is given by Eq. (16). The framework is shown in this comparison, as shown in Fig. 2, in which it is drawn in light of the prior data for an adequate sample with the given details. It involves the two equations as well. As can be seen, the NPS and NS are generally consistent with one another. Additionally, the MS shows that, up to a time of 200 units, the absolute error between the analytical and numerical outcomes is 0.0195. Moreover, the plotted waves behave periodic forms with the same number of wave, amplitudes, and wavelengths, which reveals that the obtained solutions have stable behavior and chaotic free. A good impact of various values of $\alpha \left( { = \frac{5\pi }{{20}},\frac{6\pi }{{20}},\frac{7\pi }{{20}},\frac{8\pi }{{20}}} \right)$ on the phase plane curves in the plane $uu^{\prime}$ is presented in Fig. 3. The plotted curves have a closed form, in which they are symmetric about the vertical and horizontal symmetry axes of these curves. The impression that can be considered is that the NPS has stable behavior and confirms what was previously predicted that: the solution is free from chaos.

**Figure 4:**
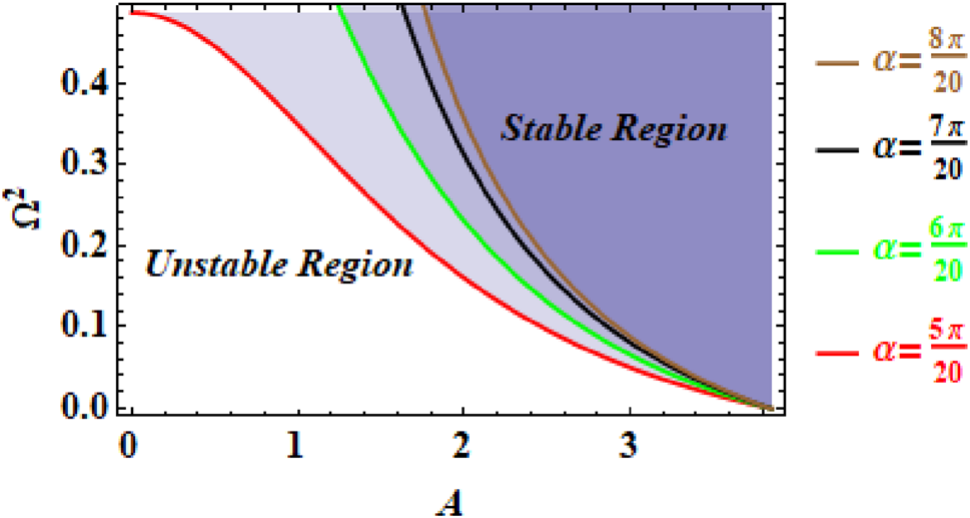
Displays the stability digram with various values of $\alpha$.

As an extra advantage of the NPS, it enabes us to discuss the stability analysis of the given nonlinear problem through handlying its alternative linear equation. Therefore, the stability profile can be sketched from analyzing the equivalent frequency, as shown in Eq. (17). Consequently, the relationship between the equivalent frequency $\Omega^{2}$ and the beginning amplitude $A$ may be drawn for various values of the angle $\alpha$. Therefore, the stable and unstable regions are calculated when $\alpha$ equals $\frac{5\pi }{{20}},\frac{6\pi }{{20}},\frac{7\pi }{{20}},$ and $\frac{8\pi }{{20}}$, as viewed in Fig. 4. As seen from this figure, the unstable regions are improved with the rise in the value of the semi-central angle $\alpha$ of the considered circular sector. Actually, this conclusion is realized.

### Second problem

**Figure 5:**
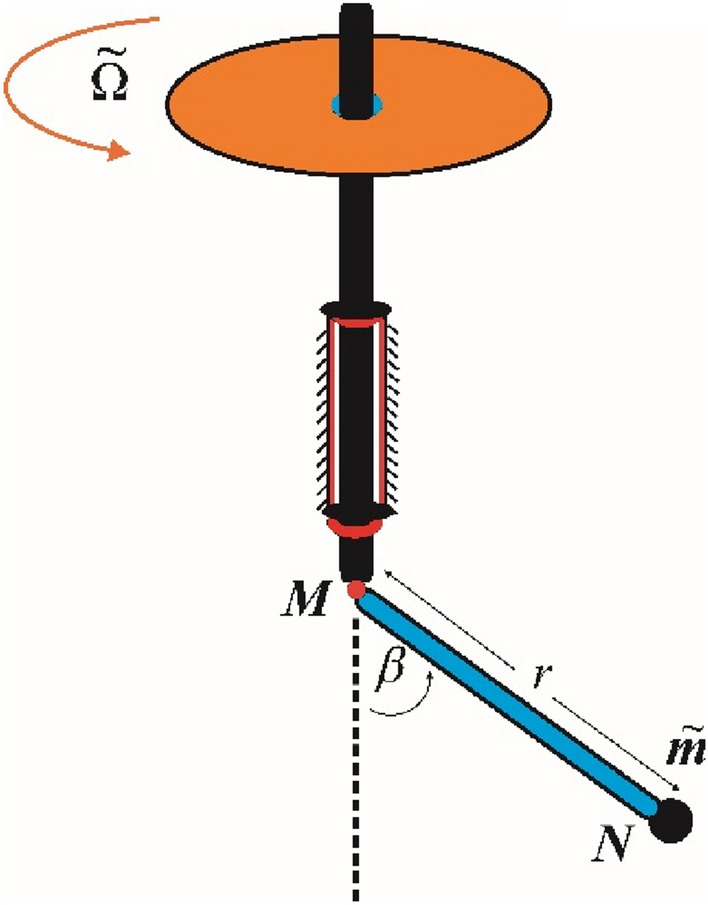
Shows a revolving simple pendulum.

The subsequent implementation, shown in Fig. 5, uses NLDEs to model the movment of a simple pendulum linked to a revolving solid framework was previously displayed^17^.

Therefore, consider the movment of a spherical pendulum as shown in the Fig. 4. The system consists of a vertical rod that rotates with a stationary angular velocity, denoted by $\tilde{\Omega }$. It is connected to the lightweight rod $MN$ through a smooth hinge. The weightless rod is connected to the mass $\tilde{m}$ at its end $N$. The objective now is to determine the equation of motion that describes the system, specifically finding the equation of motion for the mass $\tilde{m}$. This equation has been derived in detail in the research studies. It is a NLDE of first order as given below:

18 $$\frac{r}{g}\ddot{\beta } + \left( {1 - {\Lambda }\cos \beta } \right)\sin \beta = 0;\,\,\,\,\,\,\,{\Lambda } = {\tilde{\Omega }}^{2} r/g.$$

Now, let us introduce another independent variable as $\mu = \sqrt {{g \mathord{\left/ {\vphantom {g r}} \right. \kern-0pt} r}} \,t$ in Eq. (18) to obtain

19 $$\beta^{\prime\prime} + \left( {1 - \Lambda \cos \beta } \right)\sin \beta = 0;\,\,\,^{\prime} \equiv \frac{d}{d\mu }.$$

As shown in the previous problem, Ismail et al.^32^ were forced to expand the resulted restoring forces. Again, given the earlier explanation of NPM, the basic governing equation may be written as follows:

20 $$\beta^{\prime\prime} + \varphi (\beta ) = 0,$$

where the cubic Duffing function is indicated by the odd secular elements as:

$$\varphi (\beta ) = (1 - \Lambda \cos \beta )\sin \beta .$$

The corresponding linear equation can be written as follows using the comparable reasoning presented in the previous part:

21 $$y^{\prime\prime} + \sigma^{2} y = 0.$$

where the equivalent frequency is determined by a similar equation to Eq. (6).

The Mathematica software is used to specify the corresponding frequency as follows^33^:

22 $$\sigma^{2} = \frac{1}{B}\left[ {2J_{1} (B) - {\Lambda }J_{1} (2B)} \right],$$

where $J_{1} (B)$ and $J_{1} (2B)$ are the Bessel functions of different arguments.

To make things more convenient, it's intriguing to look at the connection between the corresponding linear ODE that comes from the NPM and the NS of the earlier Eq. (14) using the Mathematica software. The following figures for the chosen settings are taken into consideration as a result:

$$\Lambda = 0.1\;{\text{and}}\;B = 0.8.$$

**Figure 6:**
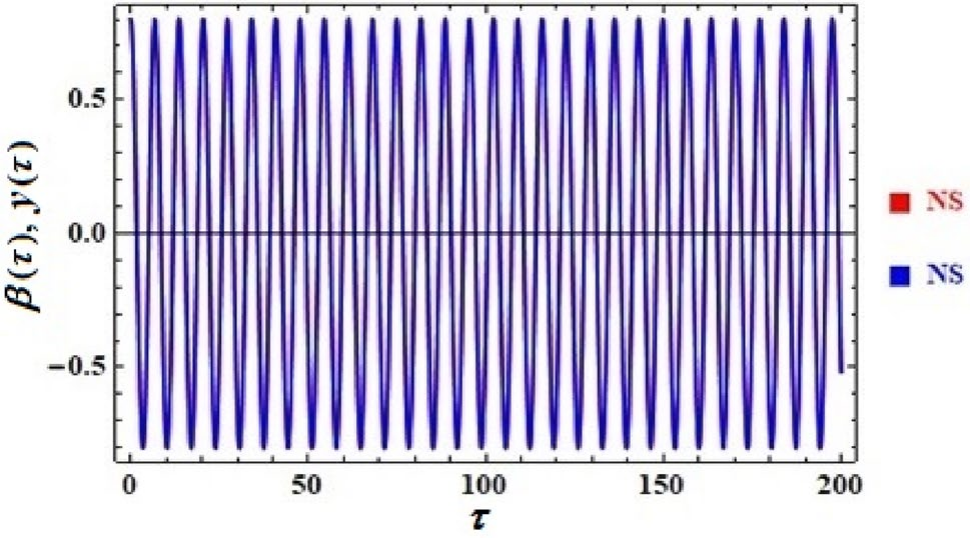
Shows an evaluation between the two solutions.

**Figure 7:**
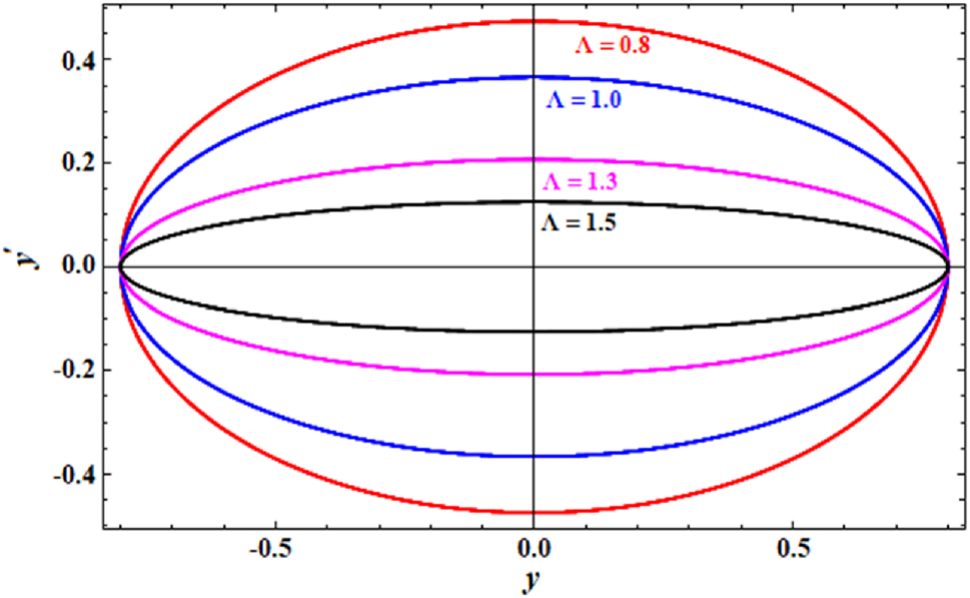
Displays the curves $y - \dot{y}$ in the plane.

It can be helpful to compare the corresponding linear ODE equation to the NS of the previous Eq. (19) that was obtained through the numerical calculation. Equation (21) contains the non-perturbative equation with frequency $\sigma$. In this comparison, the framework is depicted by the drawn curves in Fig. 6. The figure was produced using the available information for a suitable sample. The two equations are also involved. The results are typically in agreement with each other, as can be noticed. Additionally, the Mathematica programme demonstrates that the absolute disparity between the analytical and numerical outcomes is 0.041 until a specific time of 200 units. The graphed waves have a periodicity form through the investigated time interval, in which there is no change in the amplitudes of these waves or the corresponding wavelengths. This periodicity gives an impression of the steady behavior of the plotted waves. The inspection of the curves in Fig. 7 reveals that they are graphed in the plane $yy^{\prime}$ which constitutes the phase plane diagrams according to the various values of the parameter $\Lambda ( = 0.8,\,\,1,\,\,1.3,\,\,1.5)$. It is observed that the drawn closed curves seem to be symmetric about the symmetric axis, which is horizontal or vertical, to yield an impression about the steady behavior of the NPS.

The link involving the comparable frequency $\sigma^{2}$ and the starting amplitude $B$ may be constructed for various amounts of the constant $\Lambda$ in order to explore the stability assessment.

**Figure 8:**
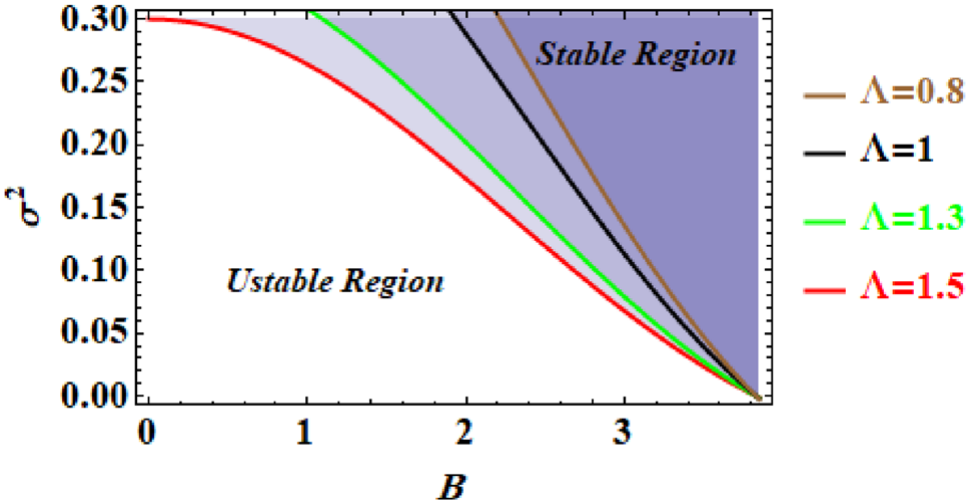
Displays the stability digram fot the second problem.

The NPS also provides us with the opportunity to address the stability examination of the provided nonlinear problem by directly manipulating its substitute linear equation. Subsequently, using Eq. (22), it is possible to draw the stability characteristic by examining the corresponding frequency. The equivalent frequency $\sigma^{2}$ is thus shown against the initial amplitude $B$. Sadly, the fundamental movement's equation as depicted in Eq. (19) only includes one parameter $\Lambda$. Based on the various values of $\Lambda$, the corresponding stability areas have been demonstrated in Fig. 8, in which the various of $\Lambda$ values depend on the parameters ${\tilde{\Omega }}$ and $r$. It can be observed from the graph that the stable zones are improved as $\Lambda$ rises. In other words, the stability profile enhances as both the constant angular frequency $\tilde{\Omega }$ as well as the length of the weightless arm $MN = r$ are enhanced.

### Third problem

**Figure 9:**
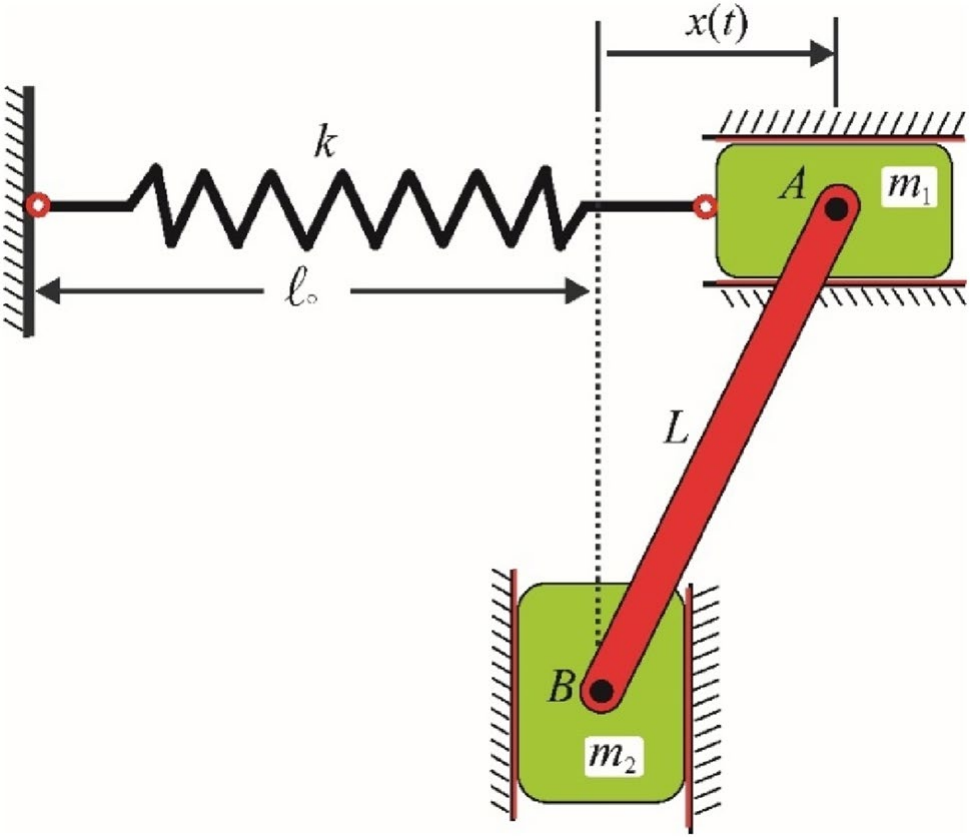
Shows the vibrating dynamical model.

The motion of a planar motion as shown in the Fig. 9 is considered. The system consists of two masses $m_{1}$ and $m_{2}$. The first mass is moved horizontally and connected to the horizontal linear spring. The other side of that spring is fixed. The rod $AB$ through a smooth hinge is connected with the first mass at $A$. The other end is connected to the mass $m_{2}$ at the point $B$.

The objective is to determine the movement's equation that describes the system. This equation has been gotten in detail in the previous work^32^. It is a NLDE of first order as given below:

23 $$\left( {m_{1} + \frac{{m_{2} x^{2} }}{{L^{2} - x^{2} }}} \right)\ddot{x} + \frac{{m_{2} L^{2} }}{{(L^{2} - x^{2} )^{2} }}\dot{x}^{2} + m_{2} g\frac{x}{{\sqrt {L^{2} - x^{2} } }} + kx = 0.$$

Now, introducing an independent variable $\tilde{T} = \sqrt {{g \mathord{\left/ {\vphantom {g L}} \right. \kern-0pt} L}} \,t$, $x = L\lambda$ in the last equation to get:

24 $$\left( {\frac{{m_{1} }}{{m_{2} }} + \frac{{\lambda^{2} }}{{1 - \lambda^{2} }}} \right)z^{\prime\prime} + \frac{{\lambda \,\lambda^{{\prime}{2}} }}{{(1 - \lambda^{2} )^{2} }} + \frac{\lambda }{{\sqrt {1 - \lambda^{2} } }} + \frac{kL\lambda }{{m_{2} g}} = 0.$$

Following further simplification as foregoing study^31^, up to the third degree in $u$, one obtains

25 $$(1 + hu^{2} )\ddot{u} + hu\dot{u}^{2} + \omega_{0}^{2} u + \xi u^{3} = 0,$$

where

$\omega_{0}^{2} = \frac{k}{{m_{1} }} + \frac{hg}{L}$ is the square of the natural frequency, $h = \frac{{m_{2} }}{{m_{1} }},\,\,\xi = \frac{hg}{{2L}},$ and $u = \frac{x}{L}$.

Given the prior discussion of NPM, the fundamental regulating equation can be stated as follows:

26 $$\ddot{u} + \phi (u,\,\dot{u},\,\ddot{u}) = 0,$$

where the odd secular characteristics as follows serve as indicators for the cubic Duffing function:

27 $$\phi (u,\,\dot{u},\,\ddot{u}) = hu^{2} \ddot{u} + Ru\dot{u}^{2} + \omega_{0}^{2} u + \xi u^{3} .$$

For more convenience, in the existing case, one assumes the initial solution as $z = D\cos \Delta \tau$.

The analogous rationale described in the preceding section can be used to write the relevant linear equation as follows^34^:

28 $$\ddot{z} + \Delta^{2} z = 0,$$

where is $z$ a new parameter which correspond the linear ODE Eq. (28) and $\Delta$ stands for the equivalent frequency.

The relevant frequency^33^ is specified using the Mathematica Software as follows:

29 $$\Delta^{2} = \frac{{3\alpha D^{2} + 4\omega_{0}^{2} }}{{4 + 2hD^{2} }}.$$

It is intriguing to examine the relationship between the equivalent linear ODE that results from the NPM and the NS of the preceding Eq. (28) employing the MS to make everything easier. As a consequence, the following numbers for the selected settings have been taken into account.

$$\xi = 0.049,\;\;\omega_{0} = 0.44,\;\; h = 0.01,\;\;{\text{and}}\;\;D = 0.1.$$

**Figure 10:**
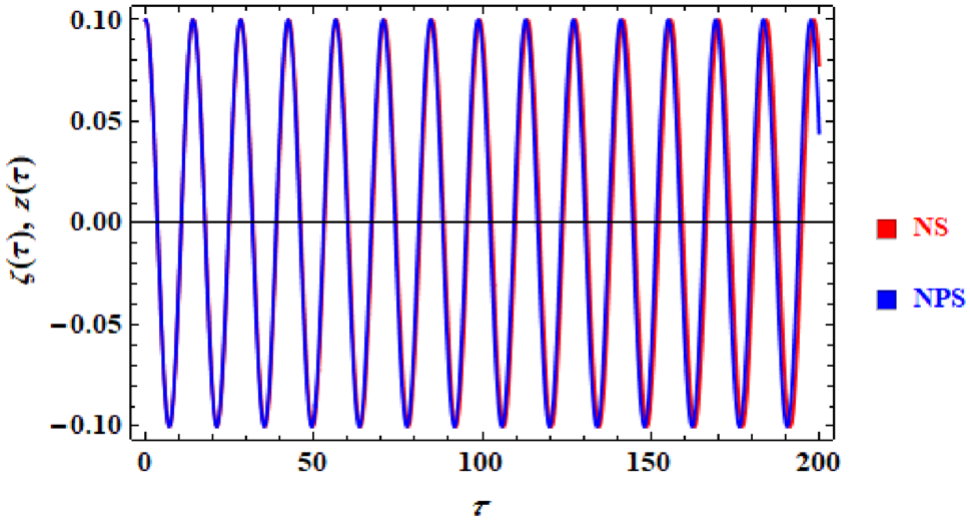
Shows a comparison between The NS and NPS.

Comparing the associated linear ODE problem to the preceding Eq. (28)'s NS, which was acquired by means of numerical computation, can be useful. The NPM equation is found in Eq. (28). Figure 10 in this comparison shows the structure. The figure was created using all of the data that was provided for an appropriate sample. There are additionally two equations involved. As can be seen, the outcomes frequently agree with one another. The depicted waves also exhibit periodic behaviour with the same number of waves, amplitudes, and wavelengths, indicating that the solutions produced have stable behaviour and chaotic-free. The Mathematica programme also shows that, until a certain point in time 200 units, the total disparity among the analytical and numerical findings is 0.01.

**Figure 11:**
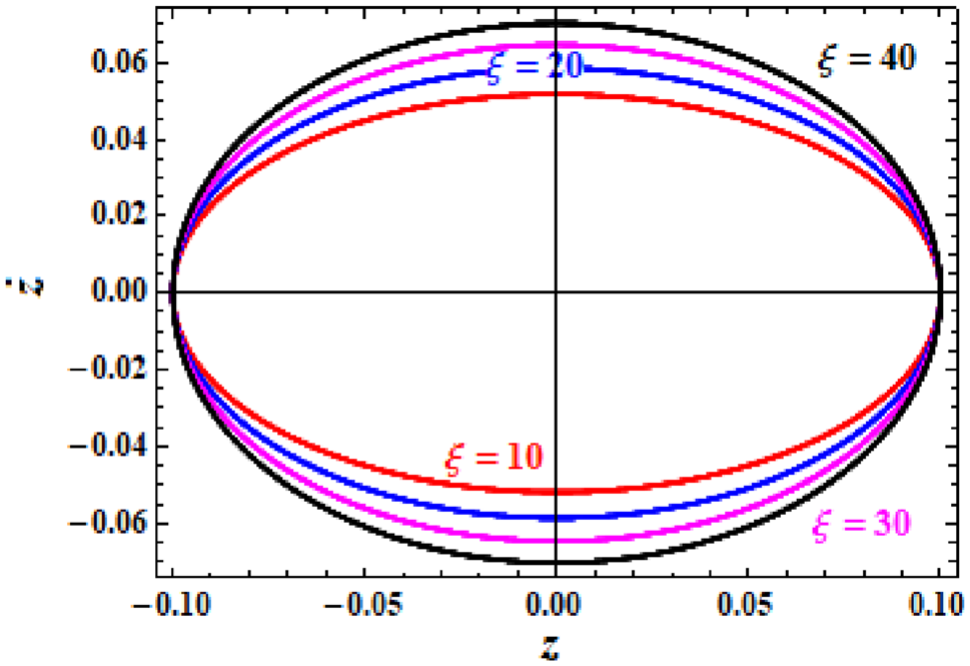
Presents the curves $z - \dot{z}$ in the plane.

The closed symmetric curves in Fig. 11 show the phase plane graphs of the NPS $z$ of Eq. (28) according to the different values of parameter $\xi ( = 10,\,\,20,\,\,30,\,40)$. These curves give an induction about the stable behaviour of the NPS.

**Figure 12:**
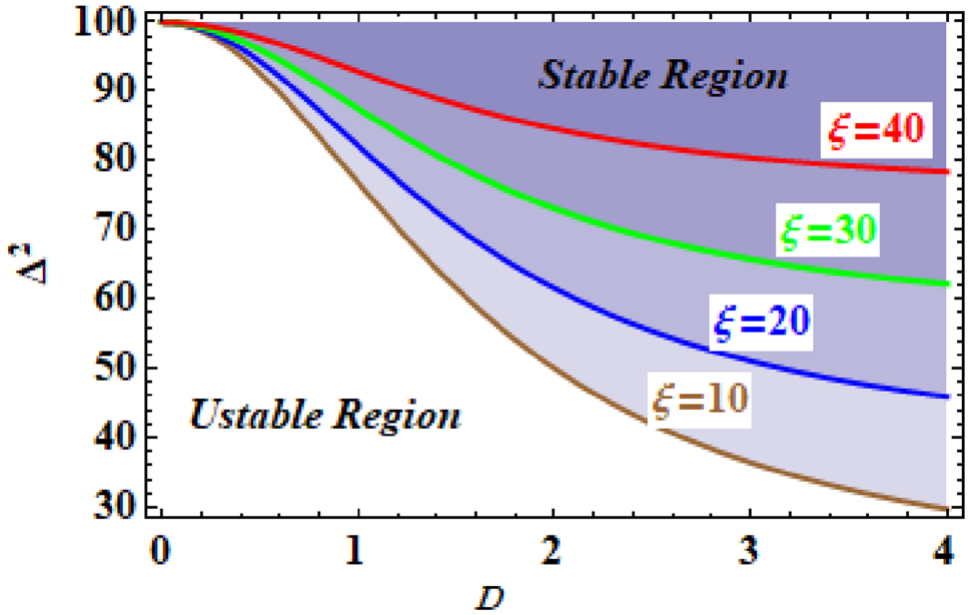
Depicts the influence of changes of the parameter $\xi$.

The relationship connecting the analogous frequency $\Delta^{2}$ and the starting amplitude *D* may be constructed for various amounts of the constants in order to discover the stability assessment. Therefore, Fig. 12 depicts the square of the equivalent frequency $\Delta^{2}$ versus the initial amplitude *D*, for various values of the parameter $\xi$. Kindly be aware that the other parameters are held fixed, where $\omega_{0} = \,10$ and $h = \,0.8$. As seen from this figure that as the parameter $\xi$ increased, the unstable zones are also increased. This shows that the parameter $\xi$ plays a role in destabilizing the stability configuration. Returning to the definition of the parameter $\alpha$, one concludes that the destabilizing zone enhances with the increasing in $m_{2}$ and decreases with both of the parameters $m_{1}$ and $L$.

**Figure 13:**
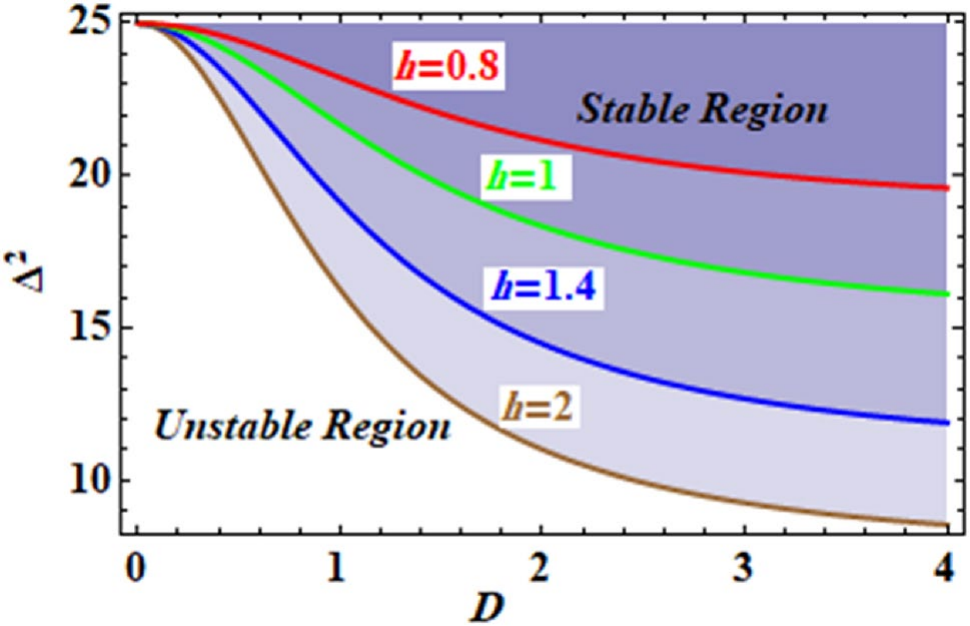
Represents the impact of the parameter $h$.

For distinct values of the constant $R$, the relationship between the equivalent frequency $\Delta^{2}$ and the initial amplitude $D$ can be graphed to determine the stability evaluation. Subsequently, Fig. 13 is plotted which shows the corresponding square of the frequency via the amplitude $D$. It is significant to note that the other factors are kept constant $\omega_{0} = 5$ and $\xi = 10$. This figure establishes how the stable zones grow larger as the parameter increases. This demonstrates how the parameter stabilises the stability organization. When one returns to the description of the parameter $h$, one comes to the conclusion that the stabilising zone expands as $m_{2}$ increases and contracts as $m_{1}$ reduces.

**Figure 14:**
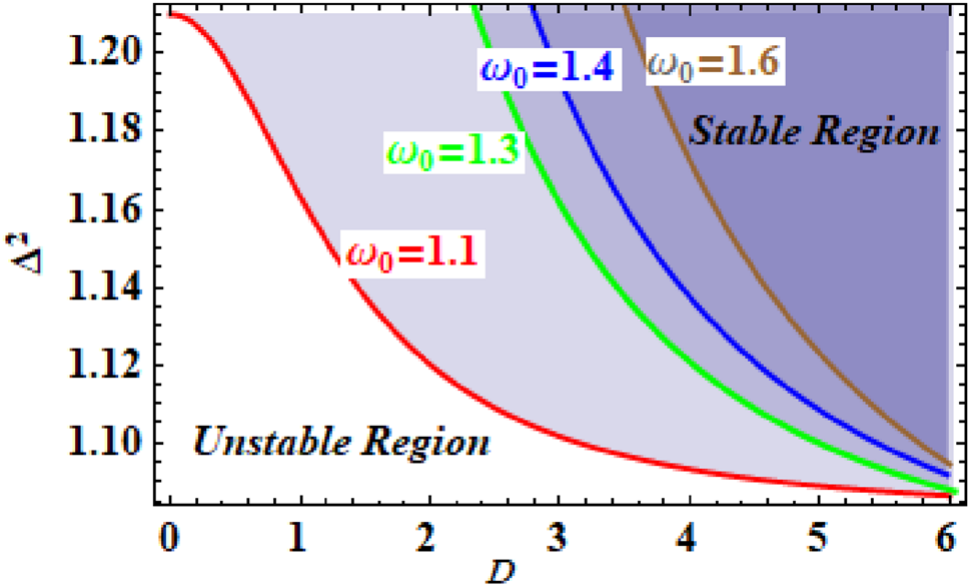
Signifies the effect of the parameter $\omega_{0}$.

The correlation connecting the comparable square of the frequency $\Delta^{2}$ and the amplitude $A$ can be constructed to obtain the stability evaluation for several values of the constant $\omega_{0}$. The corresponding frequency is then plotted versus the amplitude according to the considered values of $\omega_{0}$, as indicated in Fig. 14. The fact that the other variables remain unchanged is noteworthy, where $h = \,1.133$ and $\xi = \,0.816$. This diagram shows that the unstable zone increases as the $\omega_{0}$ rises. This exemplifies the parameter $\omega_{0}$, which indicates that the natural frequency of the system has a destabilising effect on the stability organisation. Returning to the parameter $\omega_{0}$, it is clear that the destabilising zone grows as the stiffness of the spring rises and shrinks as it decreases.

**Figure 15:**
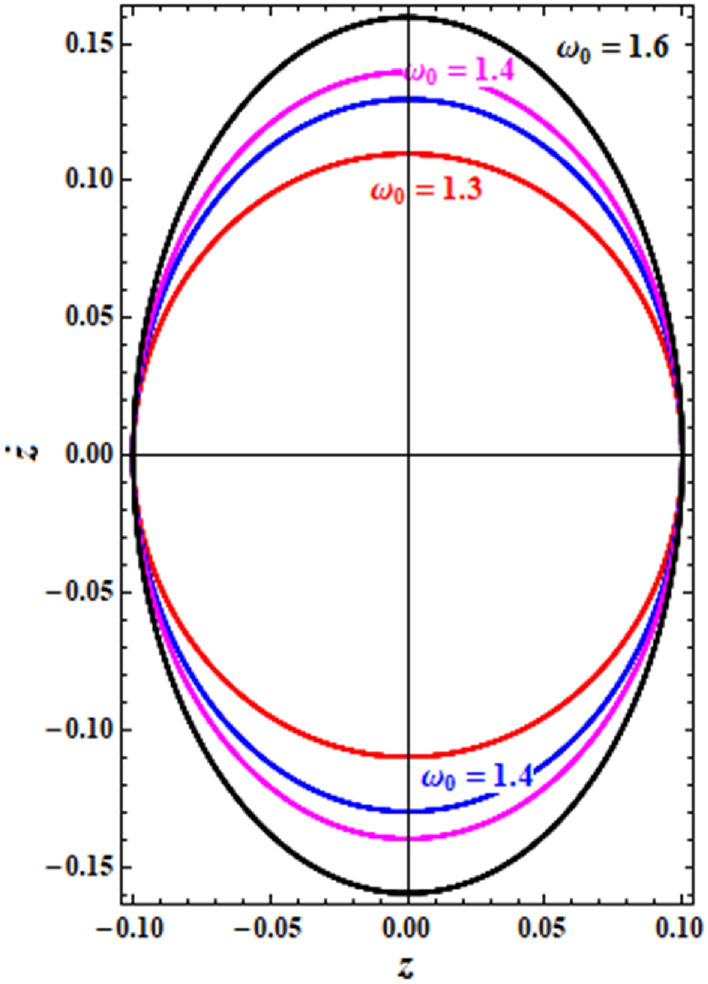
Shows the curves $z - \dot{z}$ at different vales of $\omega_{0}$.

The drawn curves in Fig. 15, represent the diagrams of phase plane for the NPS $z$ when $\omega_{0} ( = 1.1,1.3,1.4,1.6)$. These curves have closed forms that are symmetric about the symmetry axes of these curves whether horizontally or vertically.

## Conclusions

The principal objective of the current study is to utilize the fundamental He's frequency formulation to analyze analytical approximation for specific types of extremely NOSs due to the increasing interest in the field of nonlinear aspects. Through the use of three real-world instances, three different technological and scientific fields are exemplified. The new method is obviously less computationally intensive than the different perturbation approaches that already used in this area. This ground-breaking method, known as the new methodology or NPS, merely converts the nonlinear ODE into a linear one. The approach results in a new equivalent frequency that is comparable to the linear ODE. To help readers, a full description is provided to illustrate the methodology of the NPS. Theoretical outcomes are substantiated by performing a numerical comparison that achieved by the Mathematica Software. The numerical and the obtained solutions both displayed outstanding consistency. All perturbation methodologies use Taylor expansion, as well-known, when there is the presence of forces of restoration, in order to magnify these forces and thus reduce the complexity of the given problem. Under the NPS, this shortcut is no longer present. In accumulation, one may carefully investigate the problem’s stability analysis with the NPS, which wasn't feasible using earlier traditional methods. Therefore, when considering approximations of solutions for strongly NOSs, the NPS is a more trustworthy source. The NPS is a helpful tool in the disciplines of applied science and engineering because it is also easily transferable for new nonlinear problems. To support the representative relationship, it is contrasted with a numerical methodology. Concerning the utilized distinct technique or significant outcomes, it is important to emphasize the following information:

1. According to the employed method, another equivalent linear differential equation that is almost identical to the current nonlinear equation is produced.
2. For this method to be effective, two equations must exhibit a perfect match.
3. In the presence of restoring forces, the Taylor expansion is commonly employed to alleviate the complexity of the given problem. This flaw is no longer present with the used method.
4. Contrary to other conventional methods, one may employ the present method to investigate the problem of stability analysis. Therefore, the stability analysis of all three existing problem is inspected. Several diagrams are plotted to depict the influence of all parameters in the phase plane containing the equivalent frequency versus the initial condition.
5. The distinctive approach appears to be simple, valuable, and captivating tool. It can be employed for the examination of numerous NOS categories.

As a progress works, other perturbed methods as shown in Refs.^35–41^ may be adopted.

## Data Availability

All data generated or analysed during this study are included in this published article**.**

## Abbreviations

NPM: Non-perturbative methodology
ODE: Ordinary differential equation
NPS: Non-perturbative solution
HPM: Homotopy perturbation method
HFF: He's frequency formula
NLDEs: Nonlinear differential equations
NOSs: Nonlinear oscillators
NS: Numerical solution
MS: Mathematical software
DEs: Differential equations
